# Comparison of Ethanol Yield Coefficients Using *Saccharomyces cerevisiae*, *Candida lusitaniae*, and *Kluyveromyces marxianus* Adapted to High Concentrations of Galactose with *Gracilaria verrucosa* as Substrate

**DOI:** 10.4014/jmb.2002.02014

**Published:** 2020-03-20

**Authors:** Yurim Park, In Yung Sunwoo, Jiwon Yang, Gwi-Teak Jeong, Sung-Koo Kim

**Affiliations:** Department of Biotechnology, Pukyong National University, Busan 48513, Republic of Korea

**Keywords:** Bioethanol, thermal acid hydrolysis, *Gracilaria verrucosa*, enzymatic saccharification, adaptive evolution, fermentation

## Abstract

The red seaweed *Gracilaria verrucosa* has been used for the production of bioethanol. Pretreatment for monosaccharide production was carried out with 12% (w/v) *G. verrucosa* slurry and 500 mM HNO_3_ at 121°C for 90 min. Enzymatic hydrolysis was performed with a mixture of commercial enzymes (Cellic C-Tec 2 and Celluclast 1.5 L; 16 U/ml) at 50°C and 150 rpm for 48 h. *G. verrucosa* was composed of 66.9% carbohydrates. In this study, 61.0 g/L monosaccharides were obtained from 120.0 g dw/l *G. verrucosa*. The fermentation inhibitors such as hydroxymethylfurfural (HMF), levulinic acid, and formic acid were produced during pretreatment. Activated carbon was used to remove HMF. Wildtype and adaptively evolved *Saccharomyces cerevisiae*, *Candida lusitaniae*, and *Kluyveromyces marxianus* were used for fermentation to evaluate ethanol production.

## Introduction

The burning of fossil fuels increases CO_2_ levels, contributing to global warming. Fossil fuels are consumed around the world and cause environmental pollution. One of the objectives of the Korean Peninsula Energy Development Organization is the development of hydrogen energy [1]. The goals of energy research are different for each country. The United States has announced the consolidation of the bioethanol industry from 2020. Research on bioethanol is important for bioenergy production. The use of bioethanol is advantageous because it can mix with other liquid fuels such as gasoline.

The ocean covers more than 70% of the earth, and the potential of marine resources is expected to be greater than that of land resources. Therefore, marine biomass has been actively investigated for bioethanol production in recent years. Seaweeds are macroalgae that have been used as marine biomass to produce bioethanol. They are a third-generation biofuel source and grow using carbon dioxide, making them environmentally friendly [1, 2]. Seaweeds are a sustainable non-food resource with rapid biomass growth [22]. Furthermore, seaweeds do not contain lignin; thus, they are easy to hydrolyze. Seaweeds can be classified into the three categories of green, brown, and red seaweeds. In comparison with other types of seaweeds, red seaweeds have a higher sugar content, which contributes to bioethanol production. Therefore, the red seaweed Gracilaria verrucusoa was used in this study [4].

## Materials and Methods

### Raw Material

*G. verrucosa* was obtained from Wando (Jeonnam, Korea). Dried seaweed samples were ground using a hammer mill. The seaweed powder was passed through a 150-mesh sieve before pretreatment [5]. The composition of *G. verrucosa* was analyzed by the Feed and Foods Nutrition Research Center at Pukyong National University in Busan, Korea.

### Thermal Acid Hydrolysis

Thermal acid hydrolysis conditions for *G. verrucosa* were optimized by maintaining the temperature at 121°C and varying the slurry content (6.3~17.7%), nitric acid (HNO_3_) concentration (175.8~824.2 mM), and treatment time (17.58~102.42 min). The samples were transferred to a cold water bath and allowed to cool to room temperature [6]. The optimal HNO_3_ concentration was determined based on the acid hydrolysis efficiency (*E_p_*) as defined in Eq. (1):

(1) $$E_{p}=\frac{\Delta_{\mathrm{glu}+\mathrm{gal}}(g/l)}{\mathrm{TC}(g/l)}\times 100(\%)$$

where *E_p_* indicates the efficiency of thermal acid hydrolysis (%), ΔS_glu+gal_ is the increase in galactose and glucose (g/l) concentrations during hydrolysis, and TC is the total carbohydrate content (g/l) of the biomass [6, 7, 9]. Sugars in *G. verrucosa* consist mainly of glucose and galactose. Therefore, ΔS_glu+gal_ was considered as the total sugar obtained by pretreatment as shown in Eq. (2):

(2) $$Y=\beta_{0}+\Sigma_{i=1}^{3}\beta_{i}X_{i}+\Sigma_{i=1}^{3}\beta_{\mathrm{ii}}X_{i}^{2}+\Sigma_{i=1}^{2}\Sigma_{j=i+1}^{3}\beta_{\mathrm{ij}}X_{i}X_{j}$$

where Y is the response factor as sugar yield of *E_p_*, *β*_0_ is the intercept term, *β*_i_ is the first-order model coefficient, βii is the quadratic coefficient for factor i, and *β*_ij_ is the linear model coefficient for the interaction coefficient between factors i and j. The quality of fit for the polynomial model equation was expressed as the coefficient of

determination (R_2_). Response surface methodology (RSM) was utilized to optimize the condition of pretreatment with HNO_3_ and to evaluate the effect of variables including pretreatment temperature (X_1_), HNO_3_ concentration (X_2_), and reaction time (X_3_) on sugar yield (Y). The slurry was then adjusted to pH 5.0 with NaOH to measure monosaccharide content by high-performance liquid chromatography (HPLC). All statistical calculations were performed with the response surface methodology (RSM) using SAS software (ver. 9.4; SAS Institute, Cary, NC, USA) as shown in Table 1 [10].

### Enzymatic Saccharification

NaOH (10 N) was used to adjust the pH to 5.0 for enzyme activation [6, 7]. Enzymatic saccharification was conducted by adding 16 units/ml Celluclast 1.5L (854 EGU/ml; Novozymes, Bagsvaerd, Denmark) [6], 16 units/ml Cellic C-Tec2 (120 FPU/ml; Novozymes, Bagsvaerd, Denmark), and a mixture containing a 1:1 ratio of Celluclast 1.5 L and Cellic C-Tec2 (16 units/ml) [8]. Celluclast 1.5 L contains endoglucanase, and Cellic C-Tec2 is a complex of enzymes. Enzyme kinetics were determined using the Hanes-Woolf equation derived from the Michaelis-Menten equation as shown in Eq. (3):

(3) $$\frac{\left[S\right]}{V}=\frac{\left[S\right]}{V_{\max}}+\frac{K_{m}}{V_{\max}}$$

where [*s*] and *V* represent the substrate concentration (*G. verrucosa*) and the reaction rate, respectively. K_m_ is a Michaelis-Menten constant and indicates the maximum reaction rate at the substrate.

The amount of monosaccharides obtained from enzymatic saccharification was determined as shown in Eq. (4), and the efficiency was calculated:

(4) $$E_{s}=\frac{\Delta_{\mathrm{glu}}(g/l)}{\mathrm{TC}(g/l)}\times 100$$

where *E_s_* is the efficiency of enzymatic saccharification, (%), ΔS_glu_ is the increase in glucose concentration (g/L) during enzymatic saccharification, and TC is the total carbohydrate content (g/l) of the biomass. Saccharification was carried out in 100 ml of 12% (w/v) seaweed slurry at 50°C with 150 rpm shaking for 48 h. Samples were collected for the determination of monosaccharide and hydroxymethylfurfural (HMF) concentrations by HPLC [12, 13].

### Removal of HMF

HMF removal after enzyme saccharification was performed using activated carbon powder (Duksan Pure Chemical Co., Ltd., Korea). A shaking incubator was used to remove HMF produced during pretreatment and saccharification. The hydrolysate was treated with 2% (w/v) activated carbon (reaction temperature of 50°C, rotational speed of 150 rpm, and reaction time of 2 min). The adsorption surface area of the activated carbon powder was 1,400~1,600 m^2^/g. The ethanol fermentation inhibitor was removed, and the samples were centrifuged at 8,000 ×*g* for 20 min to remove activated carbon. The monosaccharide and residual HMF concentrations in the supernatant were evaluated by HPLC [5], and the HMF removal efficiency was calculated:

(5) $$E_{R}\frac{C_{\mathrm{HMF}}-R}{C_{\mathrm{HMF}}}\times 100$$

where *E_R_* is the efficiency of HMF removal (%) by activated carbon. *C_HMF_* represents the monosaccharides before HMF removal by activated carbon, and R represents the monosaccharides lost during HMF removal.

### Ethanol Fermentation

Ethanol fermentation was performed with 100 mL of 12% (w/v) *G. verrucosa* hydrolysate in 250 mL Erlenmeyer flasks. Adaptive evolution to high concentrations of galactose was carried out for 48 h, and yeasts (1.0 g dcw/l) were inoculated into 100 ml of *G. verrucosa* hydrolysate [5].

Fermentation for ethanol production was performed at 30°C and 150 rpm using yeasts that were evolutionarily adapted to galactose and wild-type yeasts with *G. verrucosa* as the substrate.

The ethanol yield coefficient (Y_EtOH_, g/g) was defined as the maximum ethanol concentration (g/l) determined based on the total initial fermentable galactose and glucose concentration at the onset of fermentation (g/l) as shown in Eq. (6) [13]:

(6) $$Y_{\mathrm{EtOH}}(g/g)=\frac{{\left[\mathrm{EtOH}\right]}_{\max}}{{\left[\mathrm{Monosaccharide}\right]}_{\mathrm{ini}}}$$

where [*EtOH*]*_max_* is the maximum ethanol concentration, and [*Monosaccharide*]*_ini_* is the concentration of glucose and galactose (g/l) at the onset of fermentation.

### Analytical Methods

The glucose, galactose, HMF, and ethanol concentrations in the samples were determined by HPLC (Agilent 1100 Series; Agilent Inc., USA) with a refractive index detector. An Aminex HPX-87H column (300 × 7.8 mm; Bio-Rad, USA) was used with filtered and degassed 5 mM sulfuric acid at an elution rate of 0.6 ml/min. Before analysis, aqueous samples were centrifuged at 8,000 ×*g* for 12 min, and the supernatant was filtered through a 0.2 μm syringe filter.

### Statistical Analysis

Optimal pretreatment conditions were determined with the RSM using SAS ver. 9.4 (SAS Institute, USA)[14, 15].

## Results

### Thermal Acid Hydrolysis

Seaweed samples were subjected to thermal acid hydrolysis. The reaction temperature and HNO_3_ concentration with various thermal hydrolysis periods were plotted based on a three-dimensional response surface method. The monosaccharide concentration was increased with the acid concentration, reaction time, and slurry concentration. Variables including the hydrolysis temperature (*X*_1_), NHO_3_ concentration (*X*_2_), and reaction time (*X*_3_) were assessed using 12% (w/v) *G. verrucosa* slurry, and the results are summarized in Table 1. The regression coefficients were calculated, and the predictive response model equation for Y as the pretreatment efficiency (*E_p_*) was expressed as Eq. (7):

(7) $$Y=-128.453973+20.677701X_{1}+0.12802X_{2}+0.355691X_{3}-0.000881X_{1}X_{2}+0.000837X_{1}X_{3}-0.000026761X_{2}X_{3}-0.717684X_{1}X_{1}-0.000108X_{2}X_{2}-0.001863X_{3}X_{3}$$

**Fig. 1:**
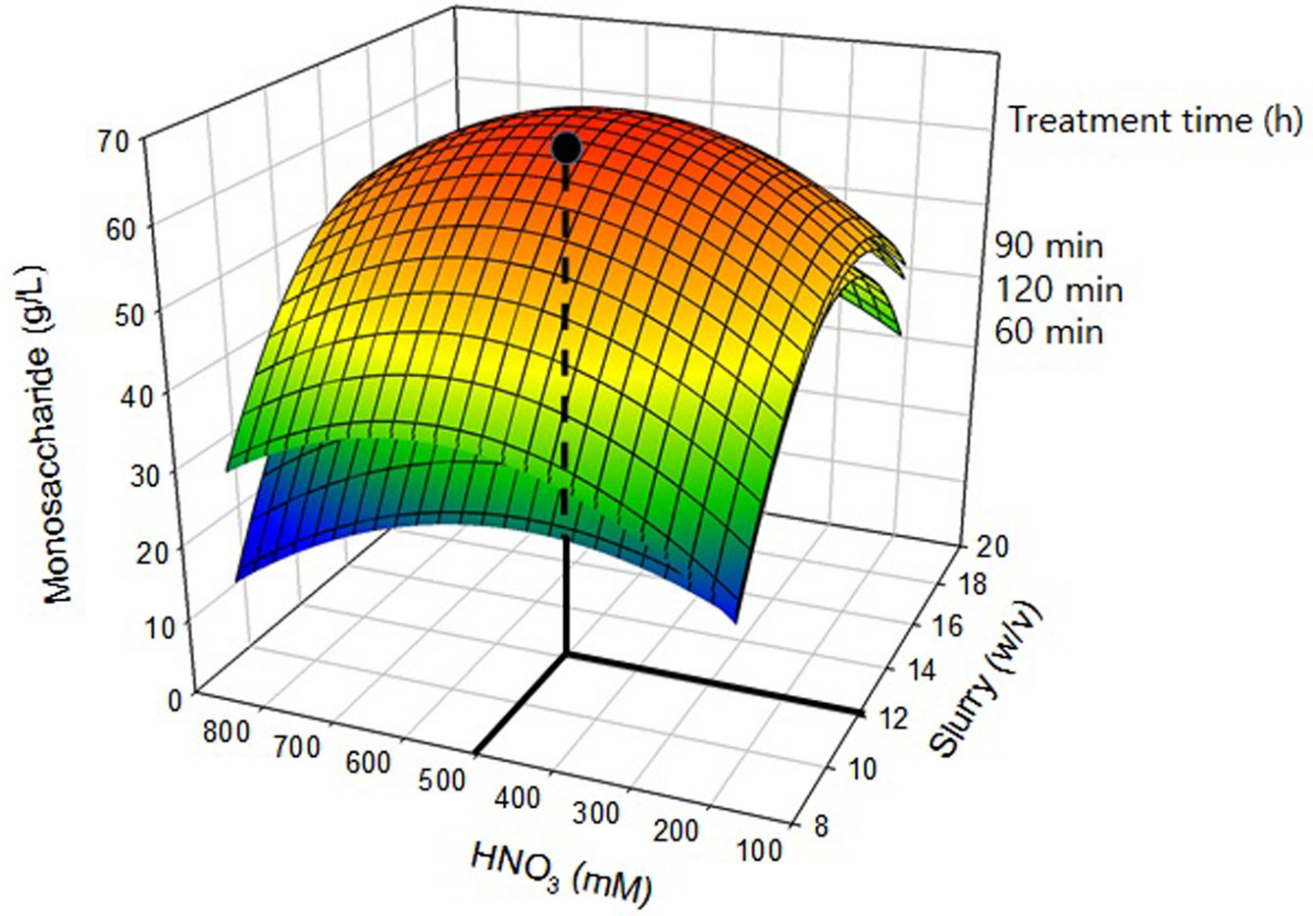
Response surface curve showing the combined effect of HNO_3_ concentration and slurry content on monosaccharide production.

Based on the high value of R_2_ = 0.9308, the regression was statistically significant, indicating that thermal acid hydrolysis had a significant effect on monosaccharide release from *G. verrucosa*. The results obtained through this equation are shown in Fig. 1. The red-colored part indicates the optimal conditions for thermal acid hydrolysis to produce 57.4 g/l monosaccharides. Green and blue areas in Fig. 1. indicate lower pretreatment efficiency. When 500 mM acid, 90 min of treatment time, and 12% slurry were used, the maximum yield of monosaccharides was obtained as shown in Fig. 1. The production of inhibitors such as formic acid, levulinic acid, and HMF occurred during acid hydrolysis. Therefore, the optimal pretreatment conditions for obtaining monosaccharides were 12%(w/v) *G. verrucosa* slurry and 500 mmol/l HNO_3_ at 121°C for 90 min.

### Enzymatic Saccharification

**Fig. 2:**
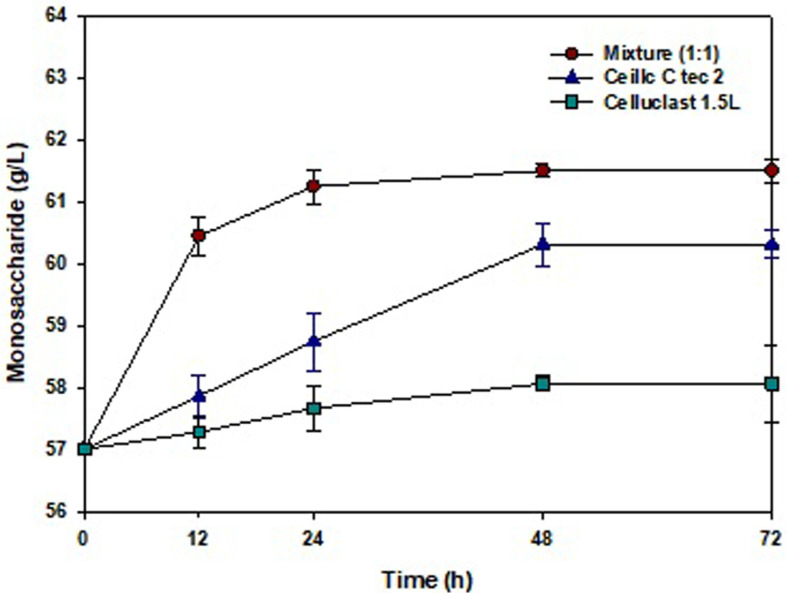
Enzymatic saccharification of *G. verrucosa* hydrolysate using a mixture of Celluclast 1.5L and Cellic C-Tec2 (1:1 ratio; 16 U/ml), Cellic C-Tec2, or Celluclast 1.5 L (16 units/ml).

Enzymatic saccharification was performed to obtain glucose after thermal acid hydrolysis [16]. Cellulase is an effective enzyme for obtaining glucose from cellulose. As shown in Fig. 2, a synergistic effect was achieved with multiple enzymes (Cellic C-Tec 2 and Celluclast 1.5 L), and saccharification was the highest compared with that of single enzyme treatments using Cellic C-Tec 2 or Celluclast 1.5 L. Therefore, enzymatic saccharification was carried out using a mixture of Cellic C-Tec 2 and Celluclast 1.5 L for 72 h. When Cellic C-Tec 2 was used as the enzyme for hydrolysis, 60.2 g/l monosaccharides were obtained, and when Celluclast 1.5 L was used, 57.8 g/l monosaccharides were obtained. When a mixture of the enzymes (Cellic C-Tec 2 and Celluclast 1.5L) was used, 61.0 g/l monosaccharides were obtained.

### Removal of HMF

**Fig. 3:**
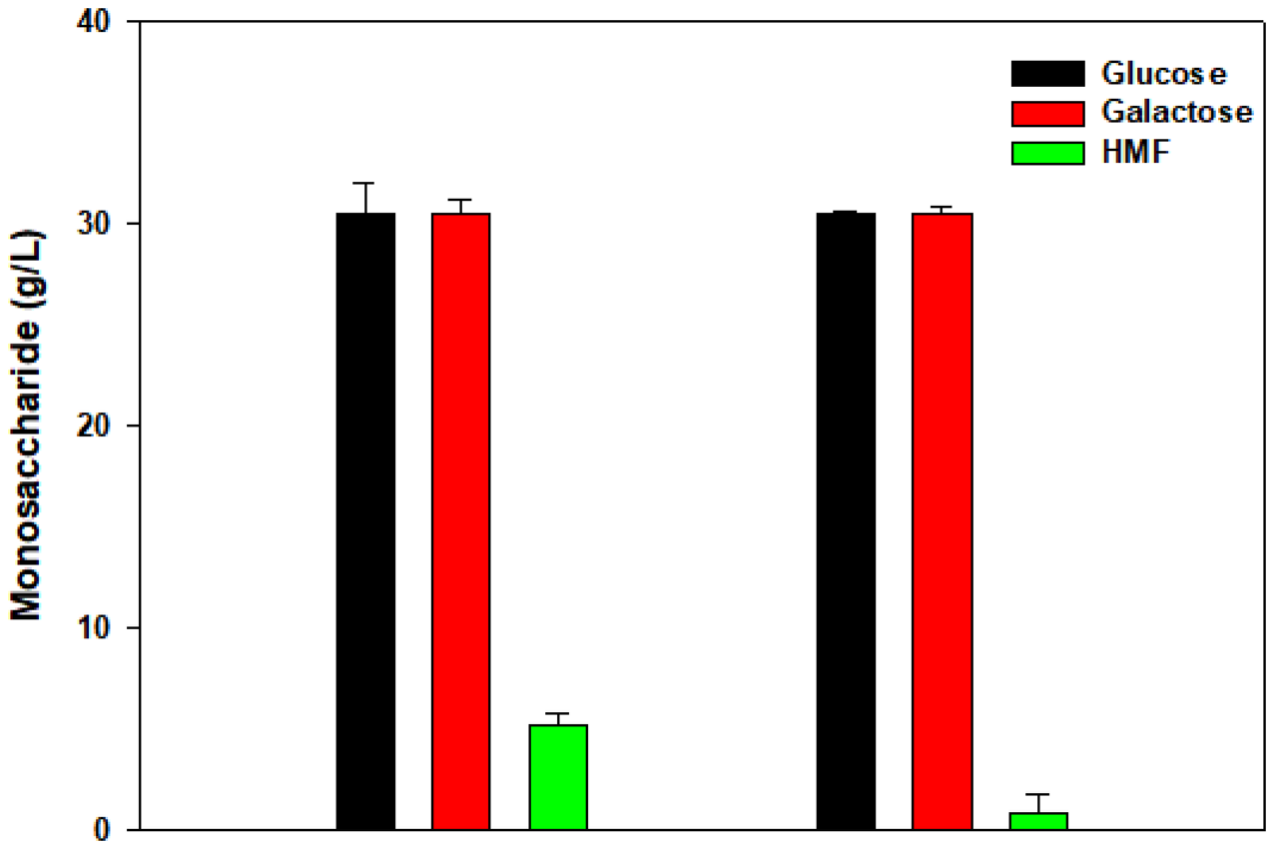
Effect of activated carbon with 2 min of adsorption time on HMF and monosaccharide concentrations in a shaking incubator at 150 rpm and 50°C (HMF removal). The bars represent the mean ± standard deviations of glucose, galactose, and HMF following thermal acid hydrolysis.

HMF is formed by the dehydration of monosaccharides and is known as an inhibitor of ethanol production. HMF was removed using activated carbon [17]. HMF removal was performed using 2% activated carbon for 2 min at a reaction temperature of 50°C with a rotational speed of 150 rpm [5]. The reaction was carried out under optimal conditions. Therefore, there was no loss of monosaccharides, and the amount of HMF was decreased from 5.1 g/l to 0.8 g/l as shown in Fig. 3.

### Fermentation

**Fig. 4:**
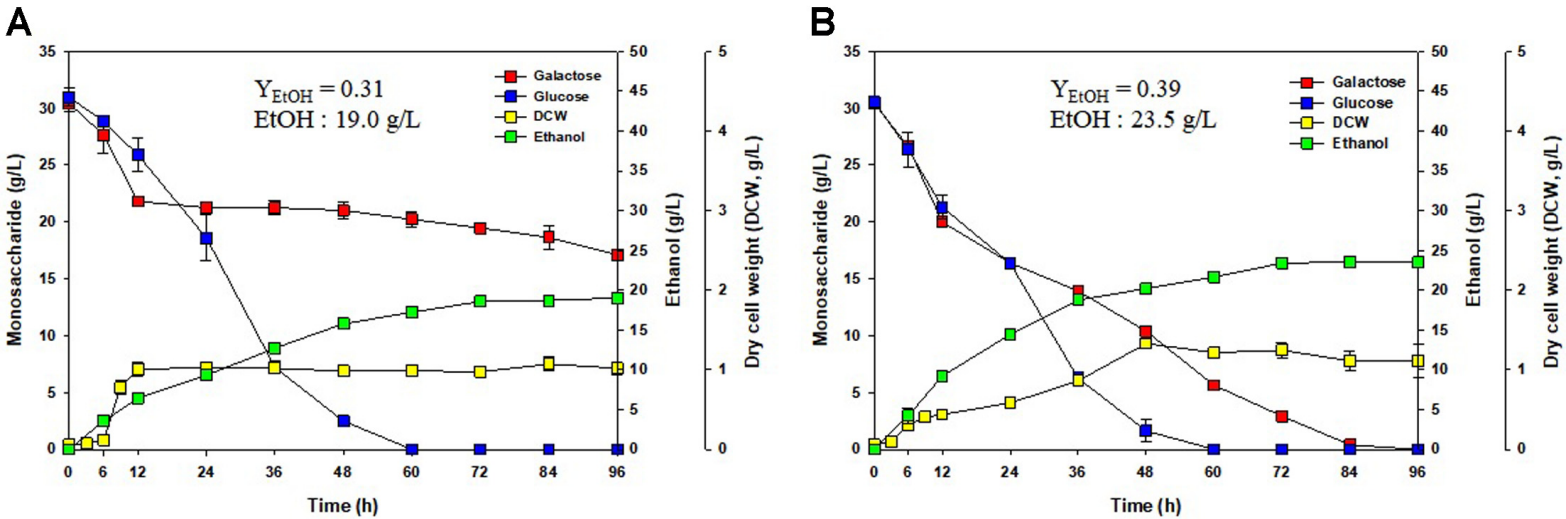
Ethanol production with *G. verrucosa* as substrate using *Saccharomyces cerevisiae* for fermentation. (**A**) Wild-type *S. cerevisiae* and (**B**) *S. cerevisiae* adapted to high concentrations of galactose.

**Fig. 5:**
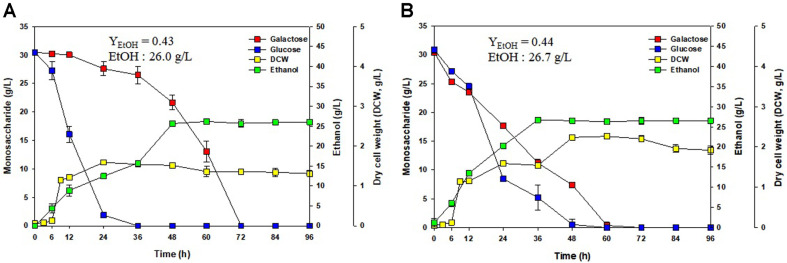
Ethanol production with *G. verrucosa* as substrate using *Candida lusitaniae* for fermentation. (**A**) Wild-type *C. lusitaniae* and (**B**) *C. lusitaniae* adapted to high concentrations of galactose.

**Fig. 6:**
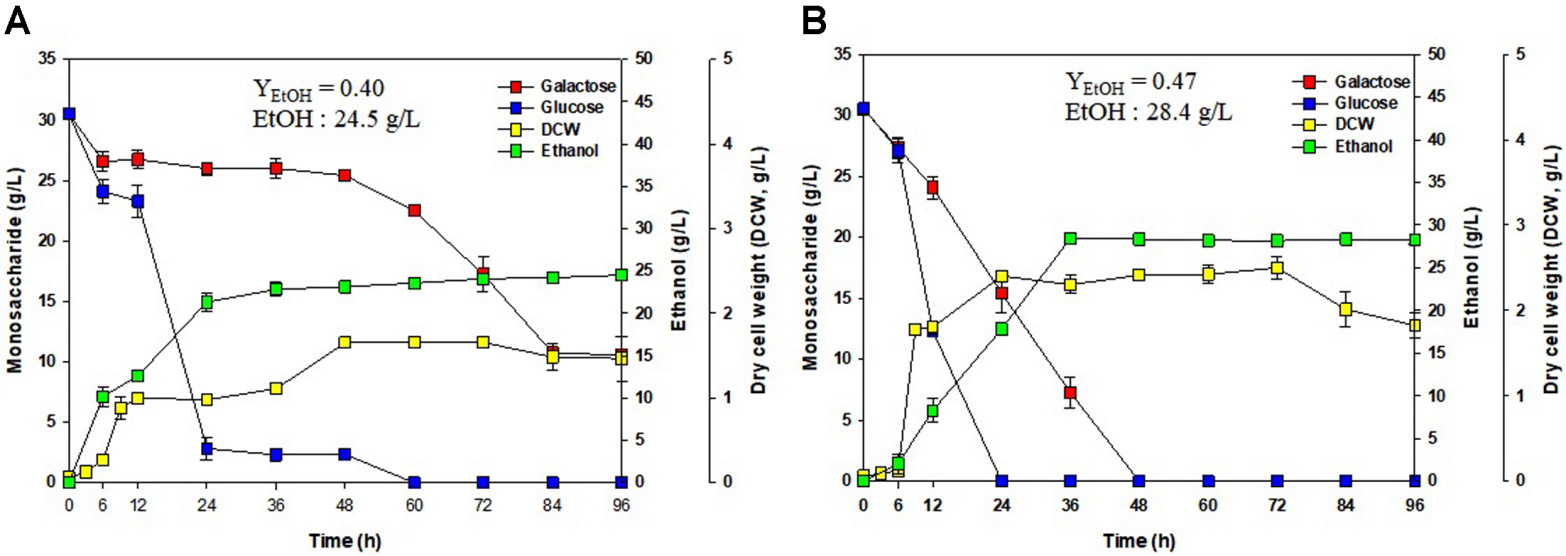
Ethanol production with *G. verrucosa* as substrate using *Kluyveromyces marxianus* for fermentation.

Three yeast strains, *S. cerevisiae*, *C. lusitaniae*, and *K. marxianus*, were used for fermentation [18]. Each fermentation was carried out with the addition of wild-type yeasts and yeasts adapted to high galactose concentrations (adaptive evolution). Ethanol was produced over 96 h of fermentation as shown in Fig. 4-6. When wild-type *S. cerevisiae* was used, ethanol production reached 19.0 g/l as shown in Fig. 4A. However, *S. cerevisiae* adapted to high concentrations of galactose produced 23.5 g/l ethanol as shown in Fig. 4B. The ethanol yield coefficient (Y_EtOH_) was 0.39 using adaptively evolved *S. cerevisiae* and 0.31 using wild-type *S. cerevisiae*. Therefore, the ethanol yield was higher using adaptively evolved *S. cerevisiae* instead of wild-type *S. cerevisiae*.

Ethanol production using wild-type *C. lusitaniae* was 26.0 g/l (Y_EtOH_ = 0.43) and using adaptively evolved *C. lusitaniae* was 26.7 g/l (Y_EtOH_ = 0.44) as shown in Figs. 5A and 5B, respectively. The use of *C. lusitaniae* adapted to high concentrations of galactose did not increase the ethanol yield coefficient significantly. This is because *C. lusitaniae* can consume galactose even if it is not adapted to high concentrations of galactose. However, adaptive evolution could reduce the time required to consume galactose. The diauxic production of ethanol was observed with wild-type *C. lusitaniae*, which exhibited a distinct pattern in the shift from glucose to galactose consumption. After complete glucose exhaustion, galactose was consumed by *C. lusitaniae*. Fig. 5A shows the complete consumption of galactose in 72 h using wild-type *C. lusitaniae*, and Fig. 5B shows the complete consumption of galactose in 60 h using adaptively evolved *C. lusitaniae*.

The outcome following the use of *K. marxianus* was similar to that following the use of *S. cerevisiae*. Wild-type *K. marxianus* produced 24.5 g/l ethanol (Y_EtOH_ = 0.40), and adaptively evolved *K. marxianus* produced 28.4 g/l ethanol (Y_EtOH_ = 0.47), as shown in Figs. 6A andespectively. Among the yeasts used in the study, *K. marxianus* produced the highest concentration of ethanol with the highest ethanol yield coefficient (Y_EtOH_ = 0.47). In addition, *K. marxianus* adapted to high concentrations of galactose produced the maximum ethanol concentration in 36 h.

## Discussion

The use of HNO_3_ produced monosaccharides in a short reaction time and showed a high saccharification efficiency compared with the efficiency using other acids such as sulfuric acid or hydrochloric acid [18, 19]. In this study, pretreatment with HNO_3_ resulted in a high monosaccharide production efficiency as shown in Fig. 1 [5]. The duration of heat treatment (60, 90, and 120 min) at 121°C was evaluated. Monosaccharide production for 90 and 120 min showed similar results. Therefore, 90 min was selected as the optimal treatment time, which was determined based on statistical evaluation using SAS software.

Monosaccharides could be obtained by thermal acid hydrolysis using HNO_3_. The optimal conditions for thermal acid hydrolysis pretreatment were 12% slurry and 500 mmol/L HNO_3_ for 90 min at 121°C. Pretreatment with *G. verrucosa* produced 57 g/l monosaccharides. The optimal conditions were used for obtaining a high concentration of monosaccharides.

A mixture (1:1 ratio) of Celluclast 1.5 L and Cellic C-Tec 2 was used in saccharification to efficiently produce glucose. The use of the two enzymes together had a synergistic effect on cellulose degradation [21]. Through the processes of thermal acid hydrolysis and enzymatic saccharification, 76% of monosaccharides were obtained from the carbohydrates of *G. verrucosa*.

A lag phase could be observed when the medium contains more than one sugar. This phenomenon, known as the diauxic production of ethanol, is caused by a shift in metabolic pathways with the change from glucose to galactose consumption as shown in Fig. 5A. After glucose exhaustion, the cells were adapted to utilize galactose. Glucose is more readily metabolized than galactose, and the presence of more readily available sugars such as glucose suppresses the synthesis of enzymes required for the metabolism of secondary sugars such as galactose. The red seaweed *G. verrucosa* contains glucose and galactose. Therefore, galactose was consumed as shown in Figs. 4B, 5B, and 6B.

For fermentation, three yeasts (*S. cerevisiae*, *C. lusitaniae*, and *K. marxianus*) were used to produce ethanol. Yeasts prefer glucose to galactose, and they have evolved to consume glucose rather than galactose. Wild-type yeasts consume minimal amounts of galactose. Therefore, the adaptive evolution of yeasts to high concentrations of galactose was performed, and the ethanol yield coefficient was improved using yeasts adapted to high concentrations of galactose. Ethanol production was increased using the adaptively evolved yeasts compared with the wild-type yeasts. Wild-type *S. cerevisiae* produced 19.0 g/l ethanol, which was increased to 23.5 g/l using *S. cerevisiae* adapted to high concentrations of galactose. Ethanol yield of 27% increase was obtained by the adaptive evolution with *S. cerevisiae*. *C. lusitaniae* adapted to high concentrations of galactose was used to increase ethanol production from 26.0 g/l to 26.7 g/l. Ethanol production by *C. lusitaniae* adapted to high concentration of galactose showed 2% increase comparing to that of wild-type strain. A significant increase in ethanol production was not observed for *C. lusitaniae* considering the active consumption of galactose by both wild-type *C. lusitaniae* and *C. lusitaniae* adapted to high concentrations of galactose. Wild-type *K. marxianus* produced 24.5 g/l ethanol, and ethanol production was increased to 28.4 g/l using *K. marxianus* adapted to high concentrations of galactose. When adaptive evolution-type *K. marxianus* was used, 16% more ethanol was produced than that of the wild-type *K. marxianus*. In contrast to *C. lusitaniae*, *K. marxianus* showed a higher ethanol production efficiency due to efficient galactose consumption following adaptive evolution. The ethanol yield coefficient was 0.40 using wild-type *K. marxianus*, whereas the ethanol yield coefficient was the highest at 0.47 using adaptive evolution-type *K. marxianus*. *S. cerevisiae* has been improved with the best adaptation effect. Adaptive evolution-type *S. cerevisiae* showed 27% higher ethanol production than wild-type *S. cerevisiae*. Among the three yeast strains, the highest ethanol yield coefficient was obtained from fermentation using galactose adaptive evolution-type *K. marxianus*.

## Figures and Tables

**Table 1:** RSM formula to determine optimal pretreatment conditions.

Design point	Independent variable			Dependent variable Y Monosaccharides (g/l)

	Slurry concentration, X_1_ (w/v)	HNO_3_ concentration, X_2_ (mM)	Thermal hydrolysis time, X_3_ (min)	
1	16	700	120	50.08478
2	16	300	120	35.71804
3	16	700	60	42.11600
4	16	700	120	50.08478
5	8	300	60	17.89879
6	8	300	120	22.63901
7	8	700	160	27.88817
8	8	700	120	30.63901
9	12	500	90	57.45969
10	17.7	500	90	53.34028
11	6.3	500	90	15.53930
12	12	824.2	90	54.27600
13	12	175.8	90	50.94499
14	12	500	132.42	56.18084
15	12	500	47.58	49.33814
16	12	500	90	57.45969
17	12	500	90	57.45969
